# Ethosomes Loaded with Cryptotanshinone for Acne Treatment through Topical Gel Formulation

**DOI:** 10.1371/journal.pone.0159967

**Published:** 2016-07-21

**Authors:** Zhenwei Yu, Hongyan Lv, Gang Han, Ke Ma

**Affiliations:** Department of Pharmacy, Sir Run Run Shaw Hospital, College of Medicine, Zhejiang University, Hangzhou, Zhejiang, People’s Republic of China; University of Helsinki, FINLAND

## Abstract

The aim of this study was to develop ethosomes loaded with cryptotanshinone (CPT) and formulate them as a topical gel for the treatment of acne. Ethosomes were prepared and evaluated for vesicle size, CPT loading and encapsulation efficiency. Optimized ethosomes were formulated as Carbomer 974 gels and compared with conventional hydroethanolic gels for transdermal permeation and skin deposition *in vitro*. The anti-acne activity and skin irritation of the gel was investigated in rabbits. Optimized ethosomes had an average vesicle size of 69.1 ± 1.9 nm with CPT loading and encapsulation efficiency of 0.445 ± 0.007 mg/mL and 40.31 ± 0.67%, respectively. The transdermal flux and skin deposition of the optimized ethosomal gel were 2.5- and 2.1-times those of conventional gels. The ethosomal gel revealed better anti-acne effect with only slight skin irritation. This study demonstrates that ethosomal formulation is an effective dermal delivery system for CPT, and that CPT ethosomal gels are promising future acne treatments.

## Introduction

Acne, a multifactorial pathology that affects skin follicles in areas such as face, upper chest and back, is the most common dermatologic disorder, affecting approximately 85% of teenagers [1]. Acne is a chronic inflammatory follicular disorder of the skin, occurring in specialized pilosebaceous units that consist of the follicular canal with its rudimentary hair and a group of sebaceous glands that surround the opening of the follicle [2]. Excess production of the male hormone androgen and colonization by *Propionibacterium acnes* have been associated in the etiology of the disease [1, 3]. Although several molecules are available for acne treatment, the majority fail to tackle all the pathogenic factors and may cause side effects including dryness, skin irritation and photosensitivity [4].

Cryptotanshinone (CPT), a well-known diterpene quinone from the widely used traditional Chinese herb *Salvia miltiorrhiza*, has been reported therapeutic potentials and diverse activities [5]. CPT is effective for the clinical treatment of acne vulgaris [6]. The mechanism of CPT in treating acne may be attributed to its anti-bacterial [7, 8], anti-inflammatory [9, 10] and antiandrogen [11, 12] activities. CPT has extremely low water solubility and an absolute oral bioavailability of only about 2% in rats [13], making dermal delivery a good choice for this compound.

Stratum corneum (SC), the outermost layer of skin consisting of corneocytes surrounded by lipid bilayers, is the main barrier for drug delivery into skin [14]. Many techniques have been used to overcome the barrier formed by SC in order to improve topical drug delivery, including ethosomes. Ethosomes are phospholipid-based nano-vesicles containing a high percentage of ethanol (20%–45%), which were developed by Touitou *et al* [15]. The ethanol in the ethosome system makes the vesicular membranes highly flexible, allowing the ethosome to transport though pores much smaller than their diameters [16]. Ethanol is also an effective permeation enhancer [17, 18]. Several studies over the past decade have extensively demonstrated the advantage of ethosomes as carriers for topical delivery and ethosomes are able to improve drug delivery into skin layers and across skin in terms of both quantity and depth [19, 20].

In the present study, CPT is loaded in ethosomes and formulated for topical delivery for acne treatment. The physicochemical properties of the ethosomes are characterized including vesicle size, CPT loading and encapsulation efficiency. The *in vitro* skin permeation and deposition of CPT in ethosomal gels are also assessed. The anti-acne effect and skin irritation are investigated in an animal model.

## Materials and Methods

### Materials

CPT was purchased from Shanxi Angsheng Biology Medical Co., Ltd (purity 98.2%, Xian, China). Soybean phosphatidycholine (SPC, Lipoid S100) was purchased from Shanghai Toshisun Biology and Technology Co., Ltd (Shanghai, China). Oleic acid and Carbomer 974 was purchased from Aladdin Chemical Reagent Co., Ltd (Shanghai, China). Polyethylene glycol 400 (PEG-400) was purchased from Hangzhou Changzheng Chemical Company (Hangzhou, China). Trypsinase was purchased from Shanghai Sangon Biotech Company (Shanghai, China).

### Preparation of CPT ethosomes

The formulations of the different ethosomes prepared are listed in Table 1. Water was slowly added in a fine stream to an ethanol solution of CPT and soybean phosphatidylcholine mixed at 700 rpm by a magnetic stirrer. Mixing was continued for an additional 15 min and then the mixture was sonicated in an ice-water bath for three cycles of 150 s using a 400 W sonotrode (2 s pulses with a 3 s interval). The mixture was passed through polycarbonate membrane filters (220 nm) three times to remove precipitated CPT.

**Table 1:** Formulation of ethosomes.

	F1	F2	F3
CPT	10 mg	10 mg	10 mg
SPC	0.4 g	0.4 g	0.2 g
Ethanol	4 ml	3 ml	4 ml
Water	6 ml	7 ml	6 ml

CPT: cryptotanshinone; SPC: soybean phosphatidylcholine

### Vesicle size and morphology

The vesicle size and polydispersity index of the ethosomes were determined in triplicate by laser scattering (Zeta sizer, Malvern Instruments, Worcestershire, UK).

### CPT loading and encapsulation efficiency

The CPT loading of ethosome suspensions were determined by dilution of samples and analysis by HPLC. The encapsulation efficiency (EE) was investigated by an ultrafiltration method. An Amicon ultra centrifugal filter unit (Amicon Ultracel 3k, Millipore, Massachusetts, USA) was employed to separate ethosomes from free CPT. Briefly, 500 μL samples of ethosome suspensions were charged to the filter unit, centrifuged at 6000 rpm for 15 min and the filtrates diluted using methanol for injected into an HPLC system for CPT quantification. The EE of ethosomes was calculated based on the following formula:

$$EE\%=\frac{CL-CF}{CT}\times 100\%$$ (1)

Where CL was the CPT loading of ethosomes, CF was the CPT concentration remaining in the filtrate and CT was the theoretical concentration of CPT. The results are expressed as the means of three independent measurements.

### Preparation of ethosomal gel

A 1% carbomer gel was obtained by adding 2 mL of 10% (w/v) sodium hydroxide to a solution of 0.5 g carbomer 974 in 48 ml water. The pH value of cabomer gel was determined in triplicate using a pH meter (PHS-3D, Jingke Corporation, Shanghai, China) after dissolving 1.0 g gel in 10 mL purified water.

The ethosomal gel was prepared by adding the ethosome suspension to 1% carbomer gel at a ratio of 1:1 (v/w) and stirring until homogeneous.

Preparation of conventional hydroethanolic gel loaded with CPT as a control; CPT equivalent to the CPT loading of the F1 formulation was dissolved in 4 mL of ethanol and mixed with 10 g of 1% carbomer gel followed by addition of 6 mL of water and mixing until homogenous.

Preparation of blank ethosomal gel; F1 formulation was prepared without CPT and blank ethosomes mixed with 1% carbomer gel at a ratio of 1:1 (v/w).

### *In vitro* study

#### Skin source

Skin was obtained from the back and abdomen of a 9- to 10-day-old pig after sacrificing by injection lethal dose of sodium barbital, which was supplied by the Experimental Animal Center of Zhejiang University. The skin was excised and prepared into uniform thickness (about 1 mm) using a drum dermatome. The prepared skin contained intact epidermis and part of dermis. The skin was stored under -80°C before use. All the procedures applied to animals in this study were approved by the Experimental Animal Use Committee of Zhejiang University.

#### Transdermal study

Vertical diffusion cells (diffusion area, 2.83 cm^2^; volume of receiving chamber, 6.8 mL) were used for an *in vitro* percutaneous study. Saline containing 40% PEG-400 was used as the receptor medium. The gel (1.0 g) was applied to the surface of the skin sample held at a temperature of 37°C with the receptor medium stirring at 250 rpm. The receptor medium (1 ml) was sampled at predetermined times up to 24 h, with the volume of each sample taken replenished by fresh medium. The CPT concentration in each sample was determined by HPLC-UV.

At the end of the experiment (24 h), the skin was washed with a cotton pad wetted with water, weighted, cut into small pieces and transferred to a plastic tube. A 1% trypsinase solution (1mL) was added and the tube incubated at 37°C for 4 h, then 4 mL of ethyl acetate was added, the mixture vortexed for 5 min and centrifuged for 10 min at 3000 rpm. The upper layer was transferred to a new tube and the ethyl acetate evaporated under a flow of nitrogen gas. The residue was reconstituted in 0.5 mL of methanol and subjected to HPLC analysis.

#### HPLC analytical conditions for CPT determination

Chromatographic analyses were carried out using an analytical Agilent HPLC unit equipped with a G1310A isopump, a 7725iManu sampler, a GA1314A UV detector and an AT-130 column heater.

The separation was performed on a C18 column (Dikma, Lake Forest, CA, USA, 150 ×4.6 mm i.d.) in the column heater set to 40°C. The mobile phase consisted of methanol:water (85:15, v:v) at a flow rate of 1.0 mL/min. The UV detector was set to 263 nm. The method had a good linearity (r2 = 0.9995) in the range of 4–1000 ng/mL with a low limit of quantification of 4 ng/mL and low limit of detection of 1.5 ng/mL.

### Anti-acne effect

An oleic acid induced acne model was used to evaluate the anti-acne effect of CPT ethosomal gels [21]. The rabbits (male, weighting about 2.5 kg) used were provided by Experimental Animal Center of Zhejiang University. Oleic acid (0.2 mL) was applied daily for 2 weeks to a 2 × 2 cm area on the underside of the pinna in the concave area just external to the ear canal. The acne model was established on 12 rabbits that were divided into 3 groups. A 0.5 g administrated of one of the three formulations (CPT loaded ethosomal gel, conventional gel and blank ethosomal gel) was applied to the acne area twice daily for 2 weeks. The rabbits were sacrificed by injection lethal dose of sodium barbital 12 hours after the last administration, the ear tissue was taken out and fixed in 4% buffered formaldehyde and routinely embedded in paraffin. Sections, 4 μm thick, were cut using rotary microtome, HM340E (Thermo Scientific, Waltham, America) and stained with hematoxylin-eosin according to guideline [22]. Briefly, the sections were dewaxed and incubated for 10 min with Harris Haematoxylin solution. Subsequently, sections were washed and stained with eosin for 2 min. The stained sections were examined using photographic microscope (Eclipse 50i equipped NIS-elements F 3.00 software, Nikon Corporation, Tokyo, Japan) at 400× magnification. The thicknesses of epidermis of each section were calculated using the equipped software. The numbers of lymphatic cell in a radon version (10 μm×10 μm) in dermis were counted.

### Skin irritation test

The skin irritation test was performed according to the OECD Guideline 404 “acute dermal irritation/Corrosion” [23, 24]. Rabbits aged 17 weeks and weighting 3–3.6 kg with healthy, intact skin were used. Hair on the back of each rabbit in an area of approximately 10 × 15 cm was shaved without damaging the skin 24 h before testing. CPT ethosomal gels or blank gels were applied to the shaved area and covered with a gauze patch held by a bandage. The patch was removed 24 h later and the skin cleaned using gauze soaked in warm water. The skin response was determined in accordance with OECD guidelines at 1, 24, 48 and 72 h. The mean scores at 24, 48 and 72 h were used to obtain the primary irritation indices. Rabbits were sacrificed after the final observation and the treated skin removed for processing and histological examination. The tests were performed on three rabbits for each group.

### Statistical analysis

Results were expressed as the mean ± SD for three or more experiments. Significance in the difference of the means was determined by the Student’s *t*-test. A value of P<0.05 was considered statistically significant.

## Results

### Characterization of ethosomes

#### Vesicle size and morphology

**Fig 1:**
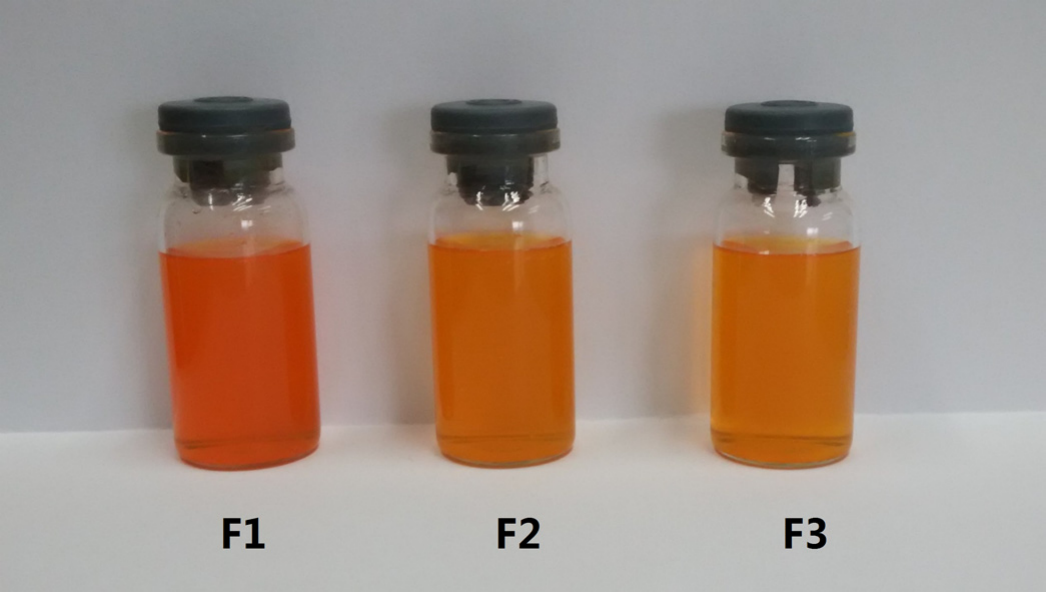
Images of cryptotanshinone loaded ethosomes.

The CPT loaded ethosomes were saffron yellow to dark red semitransparent suspensions (Fig 1). Vesicle sizes and polydispersity indexes (PDI) of each formulation are given in Table 2. The vesicle sizes of ethosomes ranged from 69.1 to 82.9 nm. The PDI were all less than 0.3, indicating that the vesicle population was relatively homogeneous in size.

**Table 2:** Characterization of ethosomes.

	vesicle size/nm	PDI	CPT loading/ mg/ml	EE/%
F1	69.1±1.9	0.185±0.017	0.445±0.007	40.31±0.67
F2	71.3±1.9	0.225±0.004	0.251±0.001	23.23±0.09
F3	82.9±2.1	0.204±0.017	0.181±0.005	9.13±0.50

CPT: cryptotanshinone; PDI: polydispersity index; EE: encapsulation efficiency

The CPT loading and EE of the three ethosome preparations are listed in Table 2. F1 exhibited the highest drug loading and EE of the three formulations used and was selected to prepare ethosomal gels.

### *In vitro* transdermal study

The pH value of blank cabomer gel was 7.21±0.01 (n = 3).

**Fig 2:**
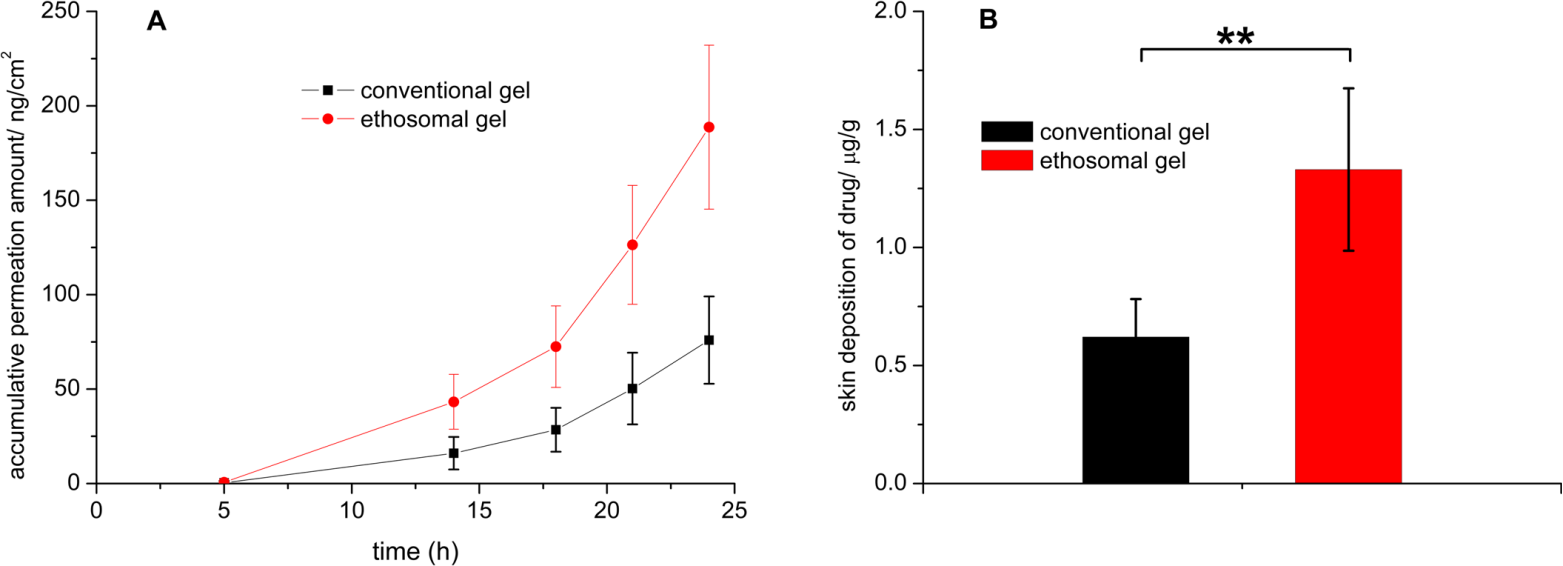
*In vitro* transdermal profiles (A) and skin deposition (B) of ethosomal gel and conventional gel, (n = 6) **, P<0.01.

The *in vitro* transdermal permeation properties of ethosomal gels and conventional gels were investigated. Transdermal permeation properties were represented by the cumulative CPT permeation per area, plotted as a function of time (Fig 2). The cumulative permeated amounts and steady transdermal flux, which were calculated from the slopes of the regression functions of the last three points of each curve, are listed in Table 3. Ethosomal gels exhibited about 2.5-times the cumulative permeation and transdermal flux of the conventional gel, which indicated ethosomes were effective dermal delivery vesicles.

**Table 3:** Transdermal parameters of ethosomal and conventional gels through excised pig skin (n = 6).

	Q_24h_/ ng/cm^2^	J_ss_/ ng/(h·cm^2^)
ethosomal gel	188.7±43.4**	19.36±4.16**
conventional gel	75.92±23.13	7.913±1.929

Q24h: accumulative permeation amount of cryptotanshinone by 24 h; J_ss_: steady permeation flux

**, P<0.01

CPT deposition into skin by the ethosomal gels was 1.330 ± 0.344 μg/g, which was 2.1-times higher than that of the control, showing that the ethosomes increase CPT deposition into skin.

### Anti-acne effect

**Fig 3:**
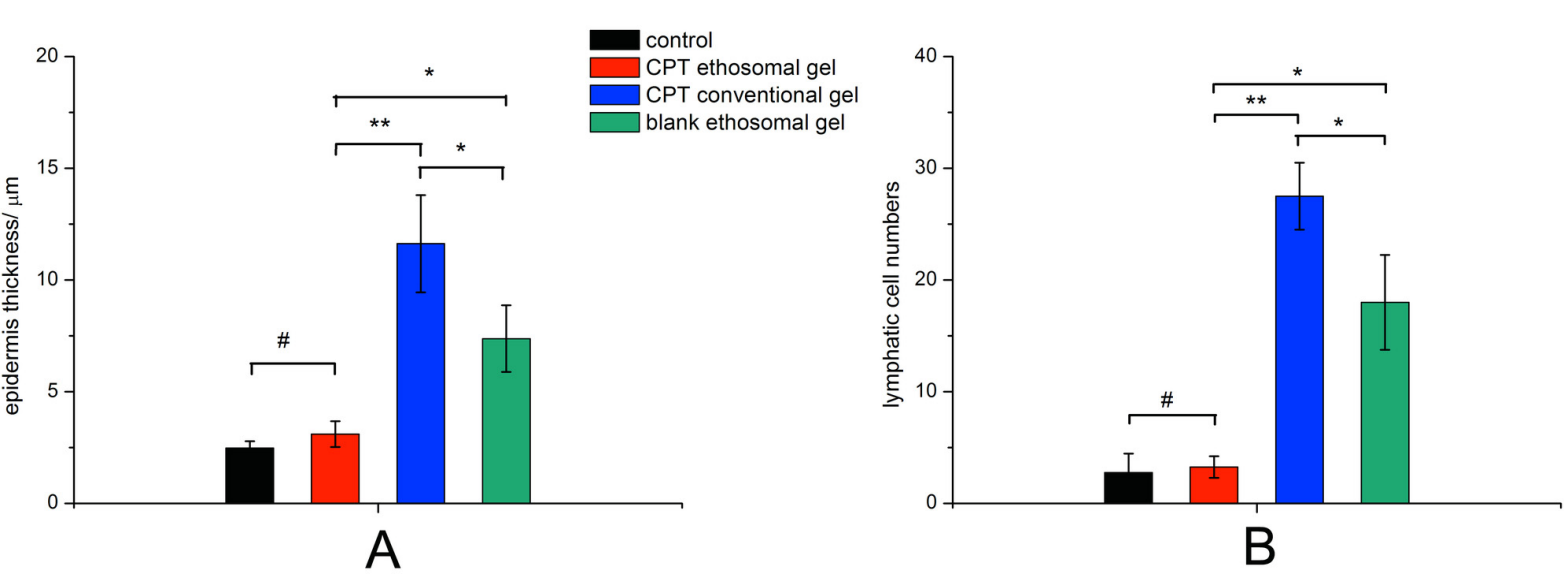
Influence of cryptotanshinone loaded ethosomal gel on epidermis thickness (A) and lymphatic cell numbers in a radon area (10 μm×10 μm) of dermis (B). #P>0.05, *P<0.05, **P<0.01.

**Fig 4:**
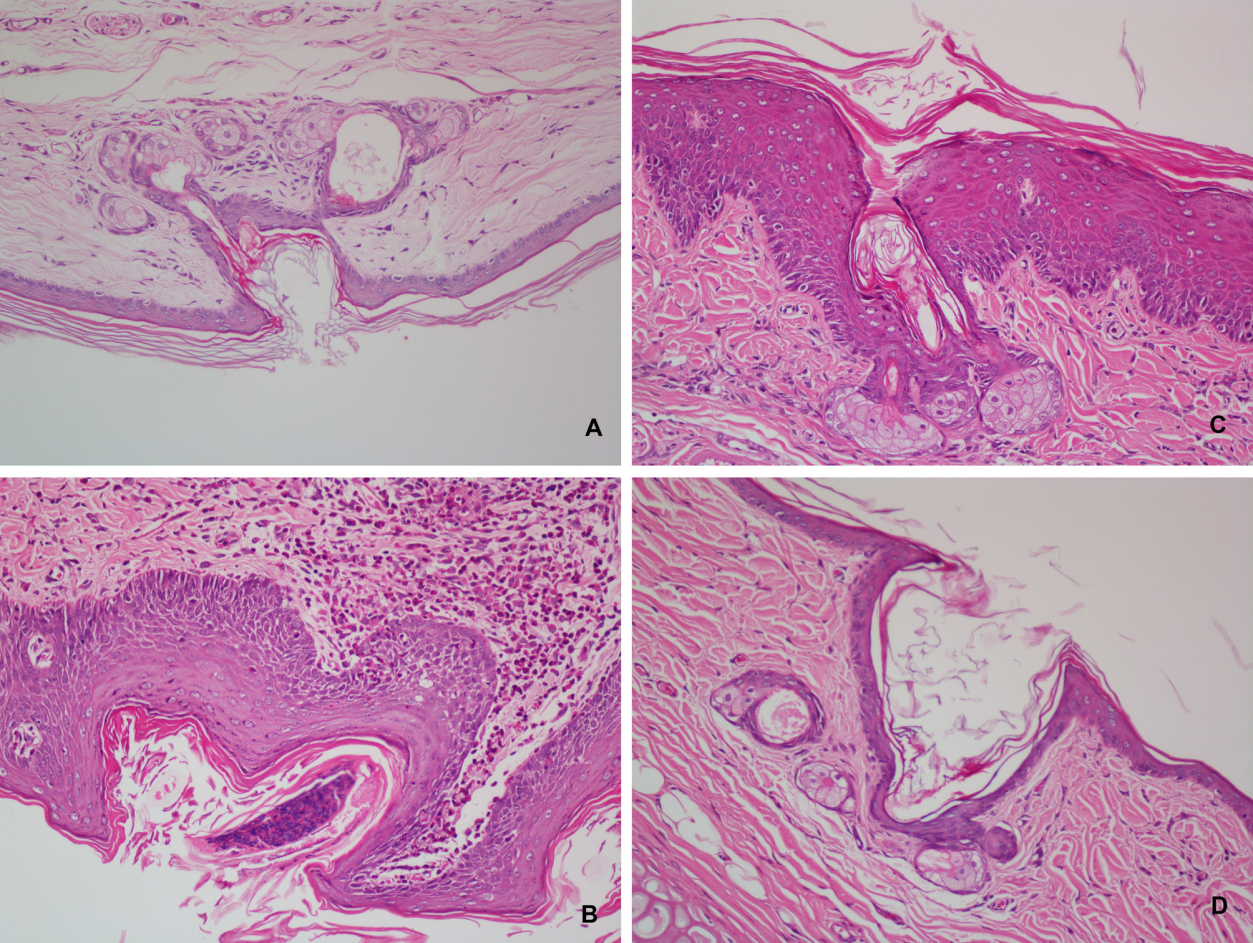
Effect of cryptotanshinone loaded ethosomal gel on the histopathology in the rabbit ear acne model induced by oleic acid. A: normal rabbit ear, B: acne model treated by blank ethosomal gel, C: conventional cryptotanshinone gel, D: cryptotanshinone loaded ethosomal gel. (400×).

The anti-acne effect of CPT loaded ethosomal gel is shown in Figs 3 and 4. Normal rabbit ear skin has thin multi-layer SC, the pilosebaceous orifice has no bacterial colonization, and both the pilosebaceous orifice and dermis have no inflammation (Figs 3 and 4A). Oleic acid induced acne model rabbits treated with blank ethosomal gel had neutrophil granulocytes and bacterial clotting in the pilosebaceous orifice, keratoplasia in the pilosebaceous orifice and SC, severe lymphatic cell infiltration in the dermis, and epidermis thickening (Fig 4B). Treatment with conventional gel prevented neutrophil granulocytes and bacterial clotting, and lymphatic cell infiltration in the dermis was obviously relieved (Fig 3B), but still existed in deep layer of skin (Fig 4C). However, keratoplasia in the pilosebaceous orifice and SC was present (Fig 4C), and epidermis thickening was sticked but not prevented (Fig 3A). The skin recovered normal structure after treatment with CPT loaded ethosomal gel (Fig 4D). There was no inflammation in the pilosebaceous unit and no keratoplasia in the pilosebaceous orifice and SC. Lymphatic cell infiltration in the dermis, including deep layers, was not found. Epidermis thicknesses had no significant difference with those of normal skin (Fig 3A). These results indicate that CPT loaded ethosomal gel had better anti-acne effects.

### Skin irritation

No visual dermal response, including erythema or edema, was found for either CPT ethosomal gel or blank gel treated groups. The primary irritation indexes of each group at each time point remained 0 and the CPT ethosomal gel has no skin irritation according to OECD guidelines.

**Fig 5:**
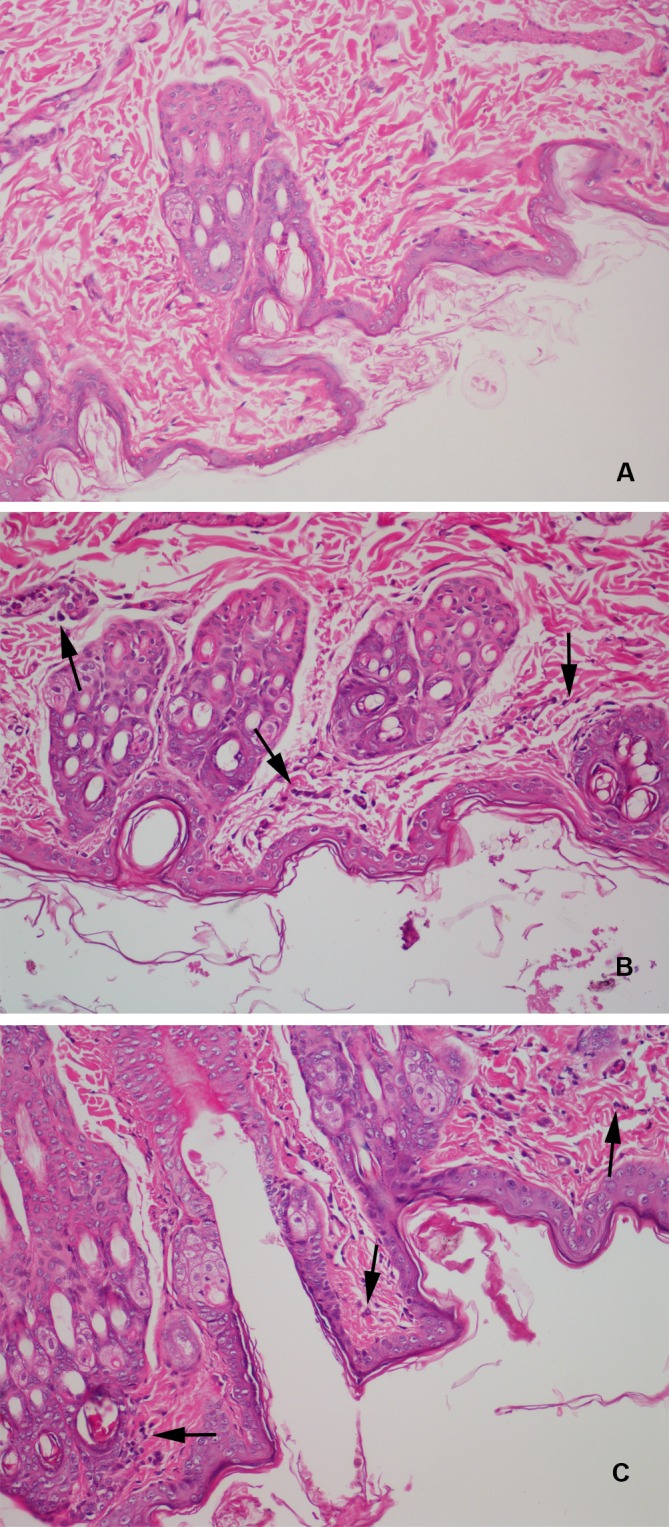
Photomicrographs (400×) of hematoxylin-eosin stained normal rabbit back skin (A), rabbit skin treated with blank ethosomal gel (B) and rabbit skin treated with cryptotanshinone loaded ethosomal gel (C).

The histological graphs of the skin structures of the CPT ethosomal gel and blank ethosomal gel groups were similar with untreated skin (Fig 5). Only a few lymphatic cells can be found in the treated rabbits (Fig 5B and 5C).

## Discussion

Vesicle size is critical to topical drug delivery systems because vesicles smaller than 300 nm are able to deliver their payloads deep into layers of the skin [25]. Our ethosomes are all below 100 nm in size (Table 2), indicating that these vesicles has potential for CPT delivery into skin. Ethanol was used because it had a fluidizing effect on phospholipid bilayers [15] and made ethosomal vesicles that are smaller in size than those of liposomes. We found that phosphatidylcholine concentration also affects the ethosome size. F1 and F2 have a similar phosphatidylcholine concentration and vesicle size, while F3 has less phosphatidylcholine and bigger sized vesicles.

Drug loading and EE are key parameters that evaluate the delivery potentiality of a system. It was repoted that dermal delivery was directed related to the drug concentration in the formulation and only entrapped drugs exhibited better permeability [26–28]. The EE of hydrophilic drug loaded ethosomes is reportedly about two orders of magnitude higher than that of liposomes [29]. This can be attributed to the greater retention of drugs in the ethanol present in the ethosomal core [30]. EE is depending on ethanol and lipid concentration. Increasing the ethanol concentration from 30% to 40% and lipid concentration from 2% to 4% increased EE because of increased fluidity of the membrane and the amount of membrane material. Ethanol concentration of more than 40% was not beneficial because it probably makes the vesicles more leaky [29]. The F1 formulation had the highest CPT loading and EE (Table 2), and was selected for further investigation.

Drugs should pass though the SC barrier regardless of whether the formulation has a systemic action or local effect. The ability of ethosomes to deliver CPT into and across the pig skin was investigated using vertical diffusion cells. CPT in ethosomal gels permeated though skin and possessed significantly higher transdermal flux than that of conventional gels (Fig 3A and Table 2). The difference in the content of CPT deposition in the skin at the end of the experiment was significantly greater when delivered from ethosomal gel compared with conventional gel (Table 2). The exact mechanism for better transfer across and into the skin for the ethosomes is still not clear. Ethanol was considered to play a major role in greater skin deposition and permeation because ethanol interacts with lipid molecules in the polar head group region. This effect leads to a reduction in the transition temperature of SC lipids, increasing their fluidity and decreasing the density of the lipid multilayer [15, 16, 31]. The ethanol concentrations in our ethosomal and conventional gels were similar. Skin deposition and transdermal flux of the ethosomal gel were greater, indicating that the ethosome structure is also responsible for the increased skin deposition and permeation.

The anti-acne effect of CPT loaded ethosomes was evaluated in gel form. Oleic acid induced acne in rabbit has been widely used as an animal model to determine the *in vivo* efficacy of anti-acne treatments [32, 33]. The histological examination of acne model skin showed inflammation, keratoplasia, bacterial colonization and epidermis thickening, which was in accordance with acne in humans (Fig 4B) [34]. CPT loaded ethosomal gels had superior anti-acne effect than conventional gels (Fig 3), and the rabbits treated with CPT loaded ethosomal gel recovered almost normal skin structure. The anti-acne effects were closely related to the trend in skin permeation and deposition of the gels. Ethosomal gel and conventional gel both relieved inflammation of the dermis, while the ethosomal gel showed better effect in the deep layers of the dermis (Fig 4). This may be attributed to the ethosomes ability to deliver CPT deep into skin layers.

Skin irritation is an important property of topical formulations. Visual inspection and scoring according to the OECD guideline showed no irritation for both CPT loaded ethosomal and blank gels. Although histological examination found lymphatic cells in the dermis of skins treated with either gel, the numbers of lymphatic cells were small and there was no other histological change (Fig 5). The CPT loaded ethosomal and blank gel have similar skin reactions, indicating that CPT itself causes no skin irritation. The skin irritation of the CPT loaded ethosomal gel can be considered as very slight and further investigation should be performed on humans to confirm the low level of irritation.

## Conclusion

CPT loaded ethosomes exhibit low vesicle size, high CPT loading and EE. The skin permeation and deposition of ethosomes formulated with carbomer gel were higher than those of a conventional hydroethanolic gel. An *in vivo* study proved that CPT loaded ethosomal gel had better anti-acne effect than a conventional gel with only slight skin irritation. This study demonstrates that ethosomes are an effective dermal delivery system for CPT, and a CPT ethosomal gel may be a viable acne treatment in the future.
